# Temperature-Induced Phase Transition in 2D Alkylammonium Lead Halide Perovskites: A Molecular Dynamics Study

**DOI:** 10.1021/acsnano.4c03903

**Published:** 2024-08-13

**Authors:** Reza Namakian, Maria Alejandra Garzon, Qing Tu, Ali Erdemir, Wei Gao

**Affiliations:** † J. Mike Walker’66 Department of Mechanical Engineering, Texas A&M University, College Station, Texas 77843, United States; ‡ Department of Materials Science & Engineering, Texas A&M University, College Station, Texas 77840, United States

**Keywords:** 2D perovskite, phase transition, molecular dynamics, order−disorder, melting

## Abstract

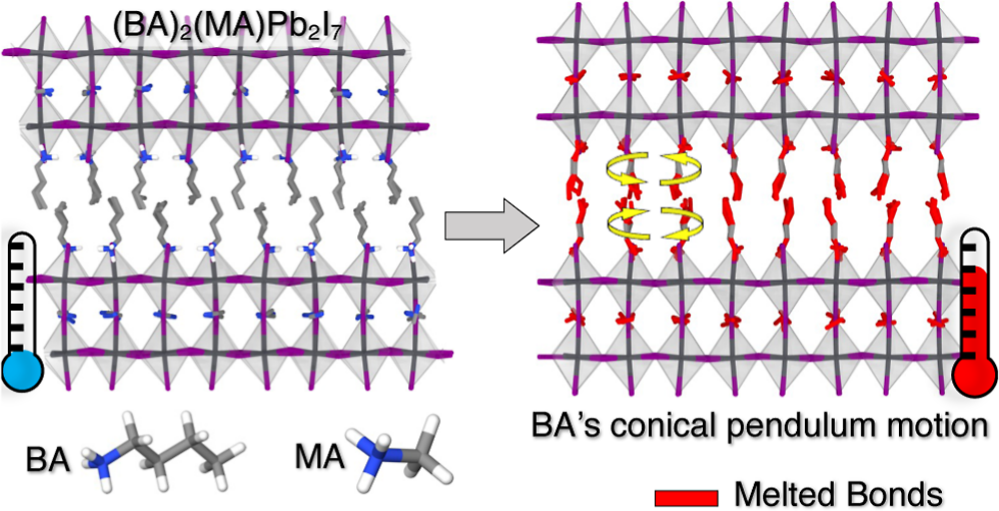

Molecular dynamics simulations are utilized to unravel the temperature-driven phase transition in double-layered butylammonium (BA) methylammonium (MA) lead halide perovskite (BA)_2_(MA)Pb_2_I_7_, which holds great promise for a wide range of optoelectronics and sensor applications. The simulations successfully capture the structural transition from low to high symmetry phases with rising temperatures, consistent with experimental observations. The phase transition is initiated at two critical interfaces: the first is between the inorganic and organic layers, where the melting of N–H bonds in BA leads to a significant reduction in hydrogen bonding between BA and iodides, and the second is at the interface between the top and bottom organic layers, where the melting of the tail bonds in BA triggers the phase transition. Following this, BA cations exhibit a patterned and synchronized motion reminiscent of a conical pendulum, displaying a mix of ordered and disordered behaviors prior to evolving into a completely molten and disordered state. While the melting of BA cations is the primary driver of the phase transition, the rotational dynamics of MA cations also plays a critical role in determining the phase transition temperature, influenced by the BA–MA interaction. Such an interaction alters the polarization patterns of MA cations across the phase transition. In particular, an antiparallel polarization pattern is observed in the low-temperature phase. Additionally, displacive elements of the phase transition are identified in the simulations, characterized by the shear and distortion of the inorganic octahedra. Notably, at lower temperatures, the octahedral distortion follows a bimodal distribution, reflecting significant variations in distortion among octahedra. This variation is attributed to an anisotropic hydrogen bonding network between iodides and BA cations. Our study reveals the phenomena and mechanisms extending beyond the order–disorder transition mechanism, suggesting potential phase engineering through strategic tuning of organic and inorganic components.

## Introduction

Metal halide perovskites, due to their excellent optoelectronic performance, have shown great promise in applications such as solar cells, light-emitting diodes, field-effect transistors, lasers, and multiple detector types, including photodetectors, X-ray detectors, and γ-ray detectors.^1^ A particular group, called hybrid organic–inorganic metal halide perovskites, has drawn much more attention due to their optical properties and solution processability. However, their tendency to degrade when exposed to ambient moisture is a significant challenge for practical applications. To tackle this problem, researchers have suggested replacing some of the organic cations with hydrophobic large organic ones. This modification led to the development of two-dimensional (2D) layered metal halide perovskites.^2−4^ With their enhanced stability and adaptable structures, they present a potential alternative to their three-dimensional (3D) counterparts.

This paper focuses on a specific layered organic–inorganic Ruddlesden–Popper hybrid perovskite, within the family of 2D alkylammonium lead halide perovskites (2D ALHPs), described by the chemical composition of (BA)_2_(MA)Pb_2_I_7_. In this composition, the halide element is iodide (I), and the divalent transition metal is lead (Pb). Methylammonium (MA) represents the MA cation with the formula (CH)_3_(NH)_3_^+^, which occupies the interstitial sites within the PbI_6_ octahedra. Butylammonium (BA), the BA cation with the chemical formula C_4_H_12_N^+^, acts as the spacer cation, separating the double-layered PbI_6_ octahedra.

Temperature-induced phase transitions of 2D ALHPs have been reported in many experiments,^5−15^ including studies on (BA)_2_(MA)Pb_2_I_7_ perovskite.^5−8^ As the temperature decreases, these perovskites experience a transition from a high-symmetry orthorhombic system to a lower-symmetry triclinic one. This reversible phase transition is characteristic of first-order order–disorder transitions, driven by the melting of the spacer alkylammonium chains. It should be noted that the terms “ordered” and “disordered” used here refer specifically to the configuration and ordering of the organic phase, not the inorganic metal-halide octahedra framework. For the double-layered LHP, Dahod et al.^6^ observed that its phase transition temperature increases as the spacer alkylammonium chain length increases, while it decreases upon substituting MA cation inside the cage with a different cation, namely, formamidinium (FA). Meanwhile, the phase transition temperature remains insensitive to the number of octahedral layers. Moreover, they hypothesized that the contraction of the organic layer might induce shearing in the inorganic layers, postulating this as a potential reason for the expanded line width observed in their powder diffractograms. On the other side, based on the observed similarities in structural dynamics between the 2D perovskite (BA)_2_PbI_4_ and the 3D perovskite (MA)PbI_3_, Menahem et al.^16^ suggested that the phase transition in 2D ALHPs is multifaceted. In addition to the melting of the BA cations, the unlocking of hydrogen (H) bonds and the subsequent anharmonic tilting of the octahedra are crucial to triggering the phase transition, suggesting a combined displacive and order–disorder mechanism for the phase transition.

Inspired by earlier findings and conjectures, our study focuses on analyzing the structural deformation and rotational dynamics of both organic and inorganic layers, including the investigation of hydrogen bonding between these layers, with the aim to understand how these factors collectively govern the phase transition process. The phenomena and mechanisms identified in our study provide valuable insights into the design principles for selecting organic and inorganic components in the context of phase transitions, aimed at enhancing the stability and performance of hybrid organic–inorganic layered perovskites at various temperatures.

## Results and Discussion

### Crystal Structure

**Figure 1:**
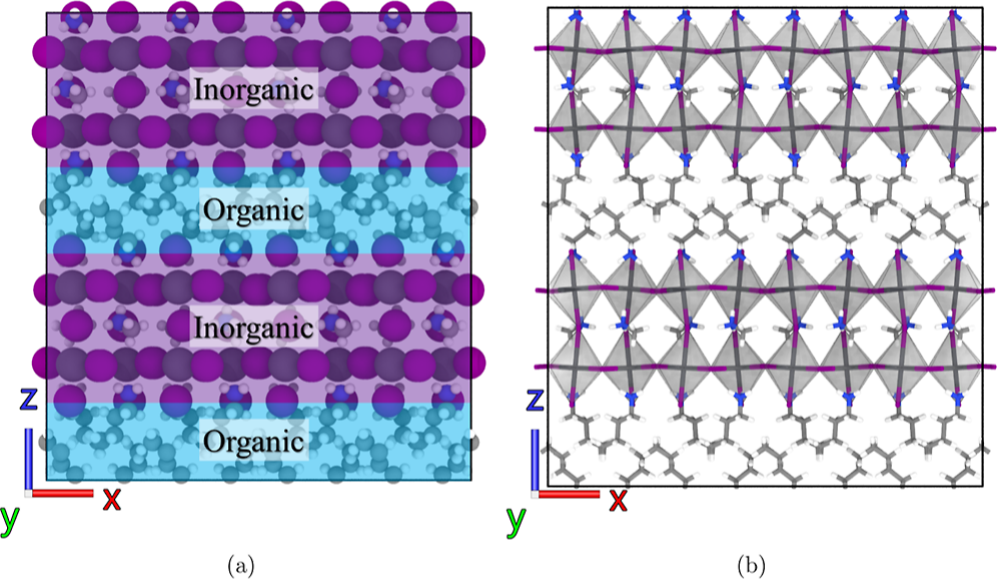
Periodic supercell of (BA)_2_(MA)Pb_2_I_7_, comprising 3264 particles. (a) Atomic structure with Pb (purple), I (dark gray), N (blue), C (light gray), and H (white) atoms. (b) Pb–I bonds in octahedra and the bonds in MA and BA, colored according to their respective atoms.

In this section, we introduce the atomic structure of (BA)_2_(MA)Pb_2_I_7_, quantify the temperature-induced structural transformation, and compare our results to experimental data. The supercell used in our molecular dynamics (MD) simulations is shown in Figure 1. The regions associated with organic and inorganic layers are highlighted in Figure 1a, where the thickness of the organic layer is determined by the time-averaged difference in the *Z* positions of iodides in the upper and lower inorganic layers, specifically those iodides separated by BA cations. The equilibrated structures are obtained at various temperatures through the annealing procedure detailed in Supporting Information (Figure S1). It is noted that the simulated annealing employed here proceeds in a stepwise fashion, with temperature intervals of 50 K from 400 K down to 200 and 25 K intervals from 200 K down to 50 K. At each temperature level, the structure is equilibrated for 30 ns at higher temperatures and 100 ns at lower temperatures. This extended sampling period is used to mitigate the effects of supercooling in MD simulations due to the limited MD time scale, aiming to capture the transition mechanism more accurately.

**Figure 2:**
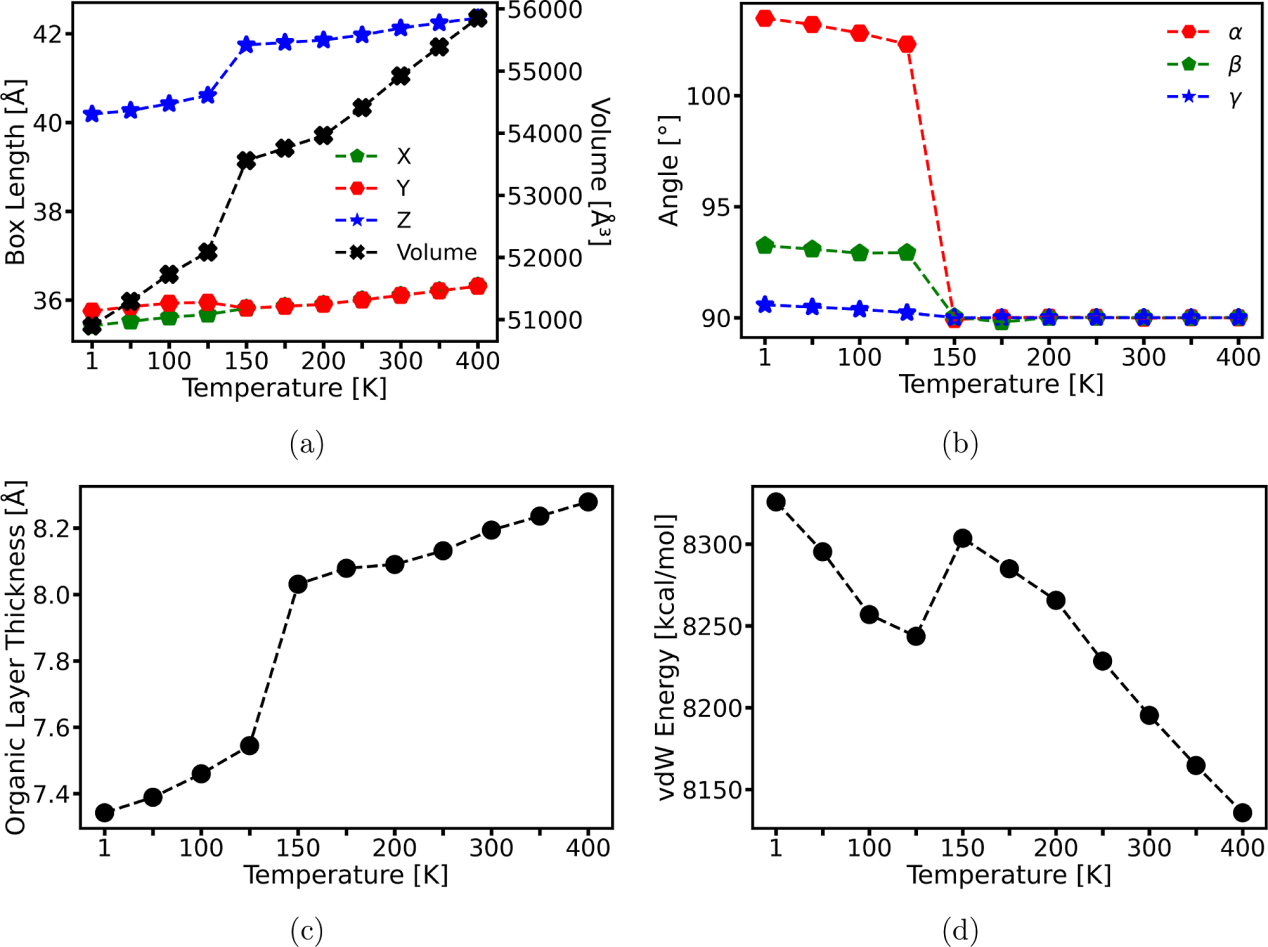
Temperature dependence of structural properties and vdW energy in (BA)_2_(MA)Pb_2_I_7_. (a) Box lengths (*X*, *Y*, and *Z*) and volume. (b) Box angles (α, β, and γ). (c) Organic layer thickness. (d) vdW energy. Data time averaged over 20 ns (400–250 K), 50 ns (200–50 K), and 4 ns (1 K) for precision.

The structural transformations, including the shape and volume of the simulation box, at various temperatures are presented in Figure 2a–c. Between 150 and 125 K, notable changes of box lengths along all three dimensions are observed, particularly a significant contraction in the *Z* direction, as shown in Figure 2a. Such contraction is primarily attributed to the organic layer, which exhibits a thickness reduction of 0.49 Å from 150 to 125 K, as shown in Figure 2c. This result is close to the experimental measurement by Dahod et al.,^6^ who reported 0.52 Å contraction in the spacing between the inorganic planes within (BA)_2_(MA)Pb_2_I_7_ during its higher to lower symmetry phase transition. In addition, Figure 2b shows that the simulation box maintains an orthogonal shape until 150 K, below which it exhibits significant angular distortions. To more precisely determine the phase transition temperature, we gradually raised the temperature from 125 to 150 K in 5 K increments after obtaining the structure at 125 K, allowing a relaxation period of 50 ns at each temperature step. No phase transition was noted until 150 K, indicating it as the critical temperature in our simulation (see Supporting Information Movie S1).

The lattice constants and angles predicted from our MD simulations are presented in Table 1, showing a low-symmetry triclinic structure below 150 K and a high-symmetry tetragonal structure at and above 150 K. These structural parameters are compared with experimental measurements for the same material,^8^ across the phase transition, showing close agreement for both phases. However, two main discrepancies are noted. First, the experimentally determined critical phase transition temperature, typically around room temperature, differs from the 150 K predicted by MD simulation. This discrepancy mainly comes from the charge assignment methodology for Coulomb interactions in the force field developed by Fridriksson et al.,^17^ which assigns formal charges of +2 and −1 to lead and iodine ions, respectively, and distributes a +1 charge across the atoms of each organic cation. This strategy was adopted for the model transferability among 2D ALHPs with different numbers of inorganic layers. However, it led to a lower phase transition temperature, even in the 3D perovskite. It is noted that the Coulombic interactions, through the formal charges implemented in the current force field, play an important role in the critical phase transition temperature in both 2D and 3D perovskites. Future research is needed to elucidate the fundamental mechanisms governing the influence of these interactions on phase transitions. Second, the high-temperature phase in the experiment exhibits a slight deviation between lattice constants *b* and *c*, suggesting an orthorhombic phase, contrary to our simulations that show *b* equals *c*, pointing to a tetragonal phase. Despite these discrepancies, it is important to note that Koegel et al.^11^ validated the force field by comparing the experimental results against the MD results generated by the same force field for 2D ALHPs. They confirmed that the force field accurately predicts the dynamics of organic cations before and after the phase transition, which is the main focus of our study. Additionally, subsequent sections demonstrate that many of our simulation results align well with experimental observations beyond just structural parameters.

**Table 1:** Average Lattice Constants and Crystal Structure Angles at Different Temperatures from MD Simulations in This Work and the Experimental Data

temperature [K]	*a* [Å]	*b* [Å]	*c* [Å]	α [deg]	β [deg]	γ [deg]
1	8.86	8.94	20.09	103.48 ± 0.01	93.25 ± 0.01	90.58
50	8.88	8.96	20.13 ± 0.01	103.20 ± 0.19	93.08 ± 0.15	90.48 ± 0.04
100	8.90 ± 0.01	8.98 ± 0.01	20.21 ± 0.02	102.81 ± 0.34	92.91 ± 0.26	90.38 ± 0.09
125	8.92 ± 0.01	8.99 ± 0.01	20.30 ± 0.04	102.31 ± 0.45	92.93 ± 0.35	90.22 ± 0.10
150	41.74 ± 0.13	8.95 ± 0.01	8.95 ± 0.01	89.99 ± 1.59	90.10 ± 1.62	90.00 ± 0.13
175	41.81 ± 0.11	8.97 ± 0.01	8.97 ± 0.01	90.01 ± 1.18	89.95 ± 1.20	90.00 ± 0.15
200	41.86 ± 0.11	8.98 ± 0.01	8.98 ± 0.01	90.01 ± 1.11	90.02 ± 1.08	90.00 ± 0.18
250	41.97 ± 0.11	9.00 ± 0.01	9.00 ± 0.01	90.02 ± 1.02	90.01 ± 1.00	90.00 ± 0.25
300	42.12 ± 0.12	9.03 ± 0.01	9.03 ± 0.01	89.98 ± 1.01	90.01 ± 1.01	90.00 ± 0.33
250^a^	8.74	9.04	19.67	102.91	95.09	91.39
300^a^	39.28	8.93	8.85	90.00	90.00	90.00

a Experimental values reported by Paritmongkol et al.^8^

In our MD simulation, we monitored variations in energy arising from different interatomic interactions, including bond, angle, dihedral, Coulombic, and van der Waals (vdW) energies. An interesting observation is that only the vdW energy displayed a pronounced jump at the phase transition temperature, as shown in Figure 2d, in contrast to other types of energy that exhibit continuous variation. The vdW energy primarily comes from the interactions between BA cations within the organic layer. It tends to increase as the temperature drops due to the decreasing distance between BA cations. At the onset of phase transition, the abrupt change in vdW energy can be attributed to the substantial structural rearrangement of the BA cations, a phenomenon further investigated in the subsequent section.

### Deformation Mechanism

**Figure 3:**
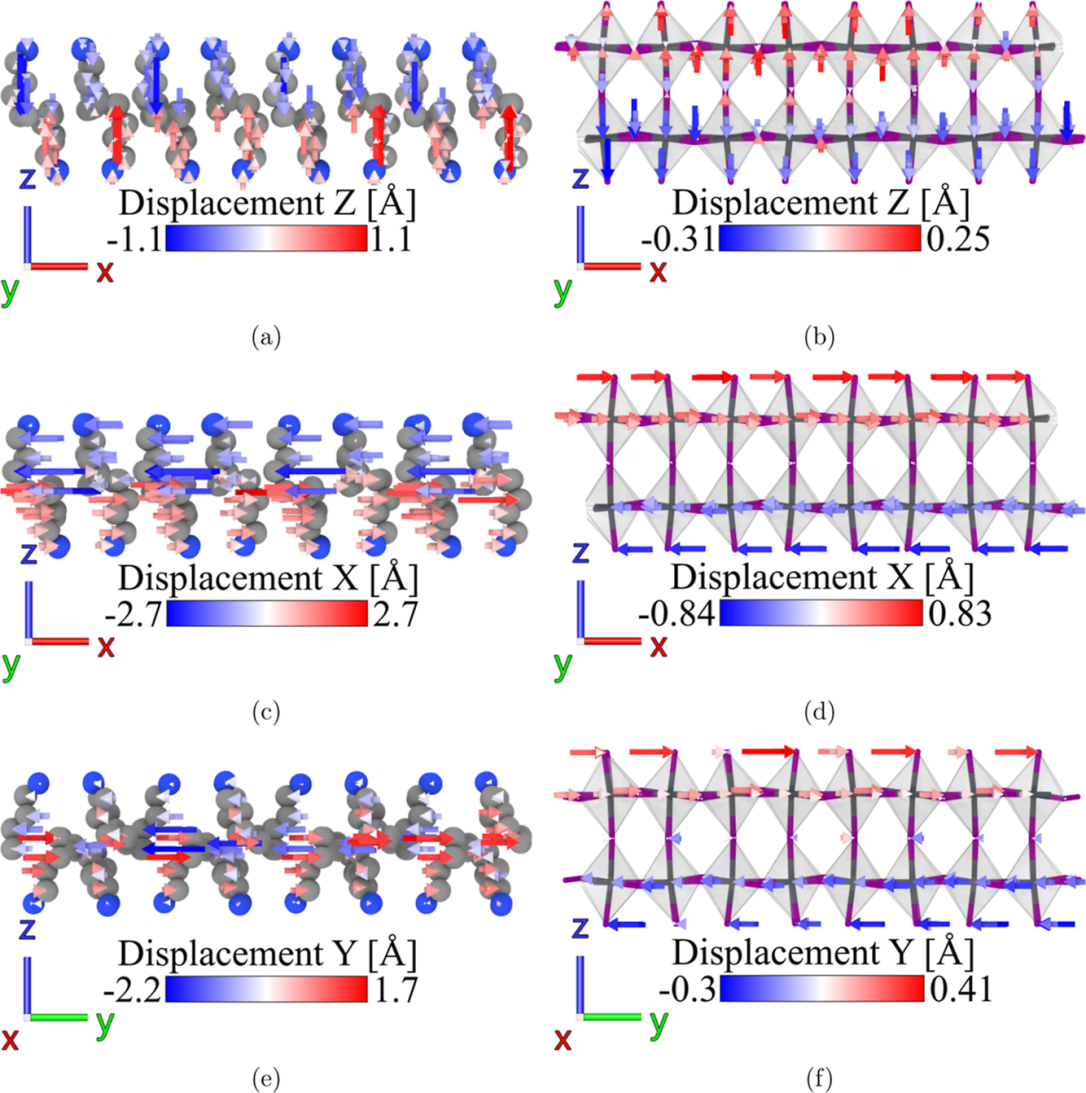
Atomic positions averaged over 200 ps within a triclinic structure at equilibrium at 125 K and atomic displacement vectors (arrows) from 150 to 125 K, scaled and color-coded based on displacement magnitude. Only BA cations’ backbone and the Pb–I skeleton of the lower inorganic layer are shown, omitting MA cations for clarity, with the Pb–I octahedra in translucent white. The left panels (a,c,e) show the *Z*, *X*, and *Y* displacement components of BA cations from different viewpoints. The right panels (b,d,f) display displacement components within the inorganic layer.

Figure 3 presents the average atomic positions of the BA cations’ backbone (N–C_1_–C_2_–C_3_–C_4_) and the Pb–I skeletons in the lower inorganic layer within the triclinic structure at 125 K. These averages were calculated over a 200 ps period in our MD simulation. The arrows plotted on the atoms represent the displacements occurring during the phase transition from 150 to 125 K. Note that these displacements exclude the macroscopic deformation of the simulation cell, and the arrows have been scaled for easier visualization.

The *Z*-axis displacements of the BA cations’ backbone, as shown in Figure 3a, correlate with the contraction of the organic layer observed during the phase transition. In addition, Figure 3c,e illustrates that the top and bottom BA cations move in opposite directions along the *X* and *Y* axes. Such shear deformation plays a key role in the tilting of the simulation box. Additionally, it results in an increased separation distance between the rearranged BA cations despite the continuous increase of interdigitation among themselves. As a result, this shear-induced rearrangement of BA cations is responsible for the reduction of the vdW energy during phase transition shown in Figure 2d. Furthermore, the displacement of the backbone atoms is observed to be nonuniform, with larger displacements at the tails. This suggests a higher mobility of the tail atoms, which correlates with the dynamics of the backbone atoms in BA cations. These dynamics and their implications are further explored in a subsequent section.

In addition to the shear deformation of BA cations, our study reveals another mechanism: shear deformation within the inorganic layer during the phase transition, as illustrated in Figure 3d,f, where the shear displacements are visualized on the Pb–I octahedra. Such shear deformation was previously hypothesized to explain the widening of line widths in powder diffractograms in experiments.^6^ Our findings not only support this hypothesis but also offer a detailed quantitative analysis of the deformation. We found that this shear deformation aligns with the shear of BA cations but with a reduced magnitude and is facilitated by significant distortion of the terminal Pb–I bonds. Meanwhile, the shear is accompanied by a slight expansion of the Pb–I skeleton in the *Z* direction, as shown in Figure 3b. Additionally, Figure 3 also shows that the octahedra experience a noticeable distortion during the phase transition. This distortion, along with the shear deformation, indicates a displacive nature of the phase transition, in addition to the mechanism of order–disorder transition within the organic layer as commonly reported in the literature. Detailed analysis of the distortion and its mechanisms is provided in a later section.

### Rotational Dynamics of BA Cations

Previous studies have revealed that the rotational dynamics of organic cations, such as MA and FA, plays a crucial role in the phase transitions of 3D hybrid halide perovskites.^18^ It was elucidated that the phase transition in 3D hybrid halide perovskites results from a combination of several factors, including a subtle interplay between dipole–dipole interactions between the organic cations, hydrogen bonding between the organic cation and the Pb–I lattice, and deformation of the Pb–I lattice in reaction to the reduced rotational motion of the organic cations. However, the role of rotational dynamics of organic cations in the phase transition of 2D perovskites is not well understood. Unlike 3D perovskites, the 2D perovskites in our study contain both the MA cation inside the octahedral cage and the BA cation within the organic layer, affecting the phase transition differently. In our study, the rotational autocorrelation function (ACF) is used to quantify the rotational degree of freedom (DOF) of the bonds of cations. In addition, the trajectories and movies from MD simulations are used to analyze the dynamic behavior. Using these two approaches, we demonstrate that the order–disorder transition mechanism, commonly attributed to the melting of BA cations, involves a more sophisticated process characterized by the changing rotation dynamics of BA cations. In the remainder of this section, we analyze the distinctive rotational dynamics of BA cations at low- and high-temperature phases, pinpointing the specific dynamics that catalyze the phase transition.

**Figure 4:**
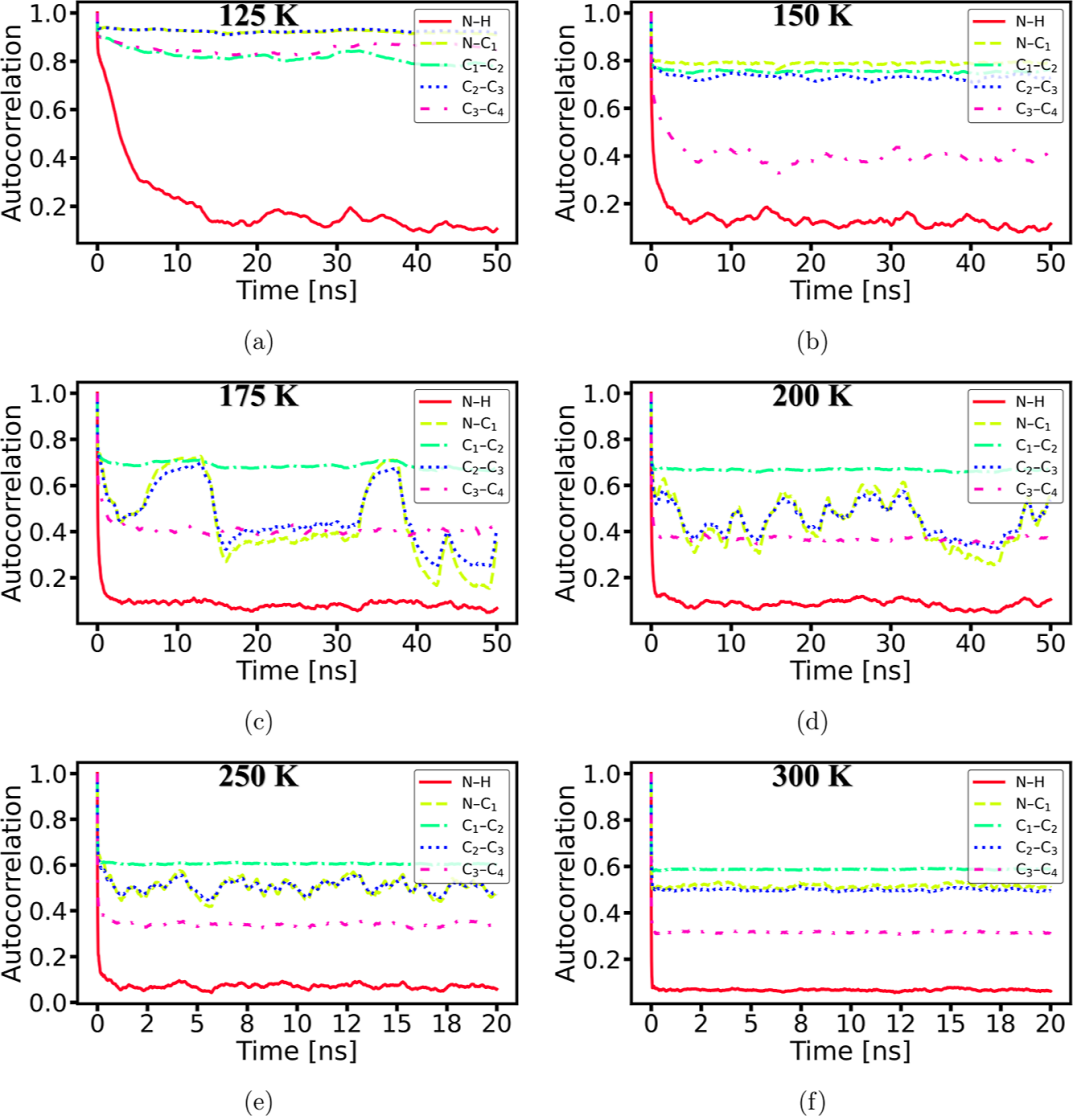
ACFs of several bonds in the BA cation’s backbone, N–C_1_–C_2_–C_3_–C_4_, and N–H bond over varying temperatures plotted against MD simulation runtime: (a) 125, (b) 150, (c) 175, (d) 200, (e) 250, and (f) 300 K.

**Figure 5:**
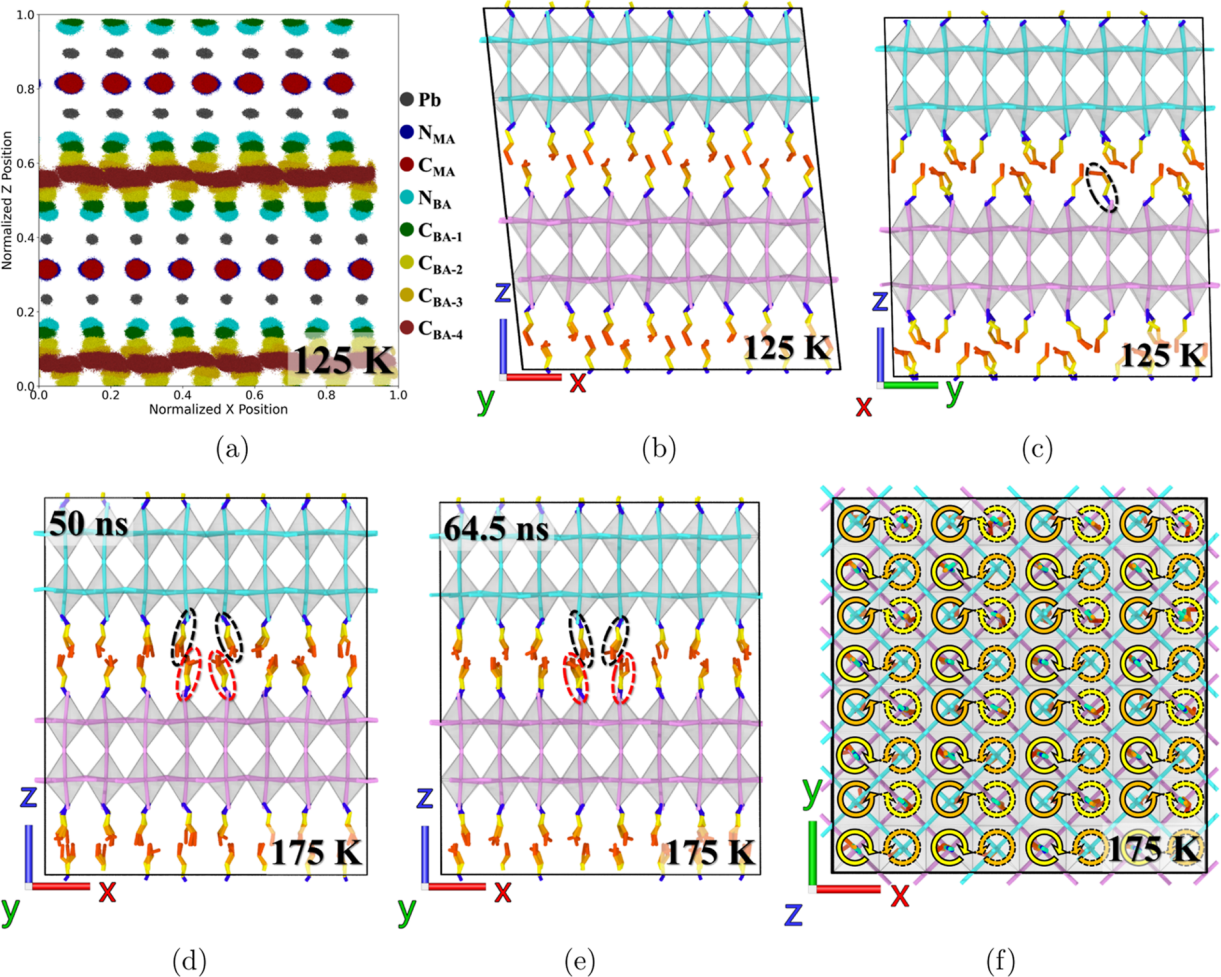
Time-averaged structures of (BA)_2_(MA)Pb_2_I_7_ at 125 and 175 K, derived from 200 ps intervals centered on selected snapshots. BA backbone and Pb–I bonds are color-coded in the respective layers, with Pb–I octahedra in semitransparent white and MA cations omitted for clarity. (a) Scatter plot of unaveraged trajectories at 125 K between 50 and 100 ns on the normalized *XZ* plane, excluding I atoms. In (c), a BA cation in trans–gauche conformation is highlighted with a dashed black oval. (f) *XY* projection of a snapshot at 175 K, illustrating instantaneous rotation directions of BA cations in the middle of the box with yellow and orange circular arrows for upper and lower inorganic layers, respectively, and a 180° phase delay indicated by dashed black outlines.

**Figure 6:**
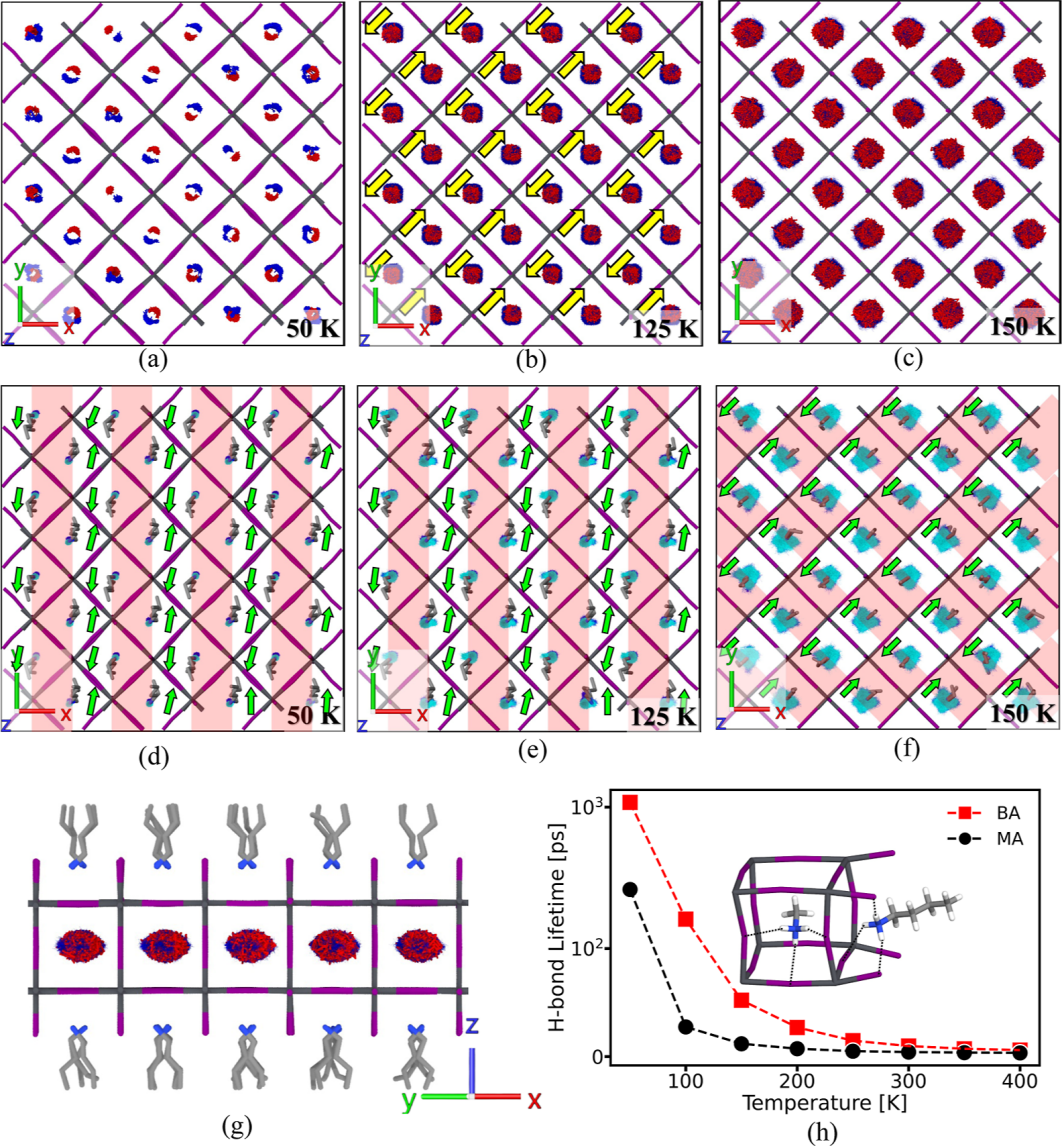
(a–c) N (blue) and C (red) in MA cations on the *XY* plane of the lower Pb–I framework over 50 ns at 50, 125, and 150 K, with yellow arrows marking the N–C bond average orientations. (d–f) N atom positions in BA cations (bottom in blue, top in cyan) and their averaged configurations, with green arrows indicating BA cation average head-to-tail orientations. Regions characterized by a higher persistence of H bonds are delineated by transparent rectangles with a red hue. (g) Trajectories of MA cations at 150 K. (h) H bonding average lifetimes between H atoms of  in BA/MA cations and I atoms in octahedra against temperature, where the embedded figure is the illustration of possible H-bond between  of BA/MA and Pb–I bonds.

The ACFs of the N–H and backbone N–C_1_–C_2_–C_3_–C_4_ bonds of the BA cation are calculated before and after the phase transition at various temperatures, as illustrated in Figure 4. A stationary bond rotation corresponds to an ACF value of 1, while a completely free rotational bond leads to an ACF value of 0. Thus, ACF can be used to track the melting of bonds during the phase transition. In the low-temperature phase at 125 K, Figure 4a shows that only the N–H bonds are melted, accompanied by significantly enhanced rotational motions of the head  groups in the BA cations. This increased mobility results in a substantial reduction in hydrogen bonding between the BA cations and iodides within the inorganic layer, indicated by an abrupt decrease in the average life of the hydrogen bonds, as further detailed in Figure 6hh. At 125 K, the backbone bonds have a much more limited rotational DOF, where C_1_–C_2_ and C_3_–C_4_ are more mobilized than the other two bonds. This limitation in rotational dynamics is consistent with findings by Koegel et al.^11^ and Lyu et al.,^9^ who reported limited rotations in BA cations below the phase transition temperatures. In addition, Figure 5a displays the projected atom trajectories from 50 to 100 ns in the MD simulation at 125 K, highlighting the most scattered trajectory for the C atom at the BA backbone’s tail, indicating its elevated mobility. As the temperature increases to the critical point of 150 K, the intensified dynamics of the tail C atom results in an abrupt drop in the ACF value for the C_3_–C_4_ bond, as illustrated in Figure 4b, indicating a melted state of the C_3_–C_4_ bond. Meanwhile, the other bonds on the BA backbone, with their much more constrained rotational DOF, remain unmelted at 150 K. Our results suggest the melting of the C_3_–C_4_ bond as a critical factor in initiating the order–disorder phase transition (see Supporting Information Movie S1). To further support this finding, we conducted an MD simulation where the rotational DOF of the C_3_–C_4_ bond was constrained. It was found that phase transition did not occur in this simulation even when the temperature was raised to 200 K (Supporting Information Movie S2).

To more accurately capture the characteristic dynamics of BA cations, atom configurations in the MD simulation were averaged every 200 ps, thereby minimizing the impact of thermal fluctuations in atom movements. In this way, MD movies (Supporting Information Movies S1 and S2–S9) were generated at various temperatures with each frame representing the averaged configuration over 200 ps interval. The movie at 125 K (Supporting Information Movie S8) reveals significantly fewer changes in atom positions compared to those observed at higher temperatures. The representative averaged configurations at 125 K are shown in Figure 5b,c. Notably, the majority of the BA cations adopt the trans–trans conformation, but some cations exhibit the trans–gauche conformation, with a representative trans–gauche example highlighted in Figure 5c. Moreover, the movie demonstrates the alternation between these two conformations. Upon cooling below 100 K, the trans–gauche conformation becomes stable and no longer alternate, as shown in Supporting Information Movie S9 and Figures S2–S4. These observations support previous experimental results, which showed the coexistence of different BA cation conformations in triclinic (BA)_2_(MA)Pb_2_I_7_ under low pressure.^19^

In the high-temperature regime, the ACF of the bonds N–C_1_ and C_2_–C_3_ in the BA cation’s backbone shows synchronized oscillation below 300 K in Figure 4, indicating a distinct dynamic behavior of the BA cations in the high-temperature phase. This dynamics is visualized by projecting the motion of the BA cations into the *XZ* plane in Figure 5d,e, which displays the time-averaged configurations at 50 and 64.5 ns from MD simulations. The BA cation’s backbone displays an interesting pendulum-like movement, as highlighted in the figure. Supporting Information Movies S4–S6 illustrate this motion in three dimensions as a conical pendulum-like movement. Moreover, this movement is characterized by two notable features. First, the BA cations connected to the top and bottom octahedra rotate in opposite directions—clockwise and counterclockwise, respectively—as shown in Figure 5f, where the rotation directions are marked with circular arrows within the projected octahedral cages. The yellow color represents the rotations of BA cations connected to the top layer, and the orange color represents those attached to the bottom layer. Second, the adjacent BA cations, on either the top or bottom layer, display an azimuthal phase difference of approximately 180°, differentiated by solid and dashed lines in Figure 5f. This synchronized conical pendulum-like dynamics vanishes as the temperature increases to 300 K, as shown in Supporting Information Movie S3, at which the BA cations are completely melted to a fully disordered phase.

The dynamic analysis of BA cations can be summarized as follows. BA cations are in an ordered state in the low-temperature phase. As the temperature increases, the melting of N–H bonds leads to a significant reduction in hydrogen bonding between the headgroup of BA and the iodides. Meanwhile, melting of the tail bond of BA triggers the phase transition. With further temperature increases, the enhanced rotational DOF, particularly in the bonds N–C_1_ and C_2_–C_3_, leads to a partially ordered and synchronized conical pendulum-like dynamic behavior. At this stage, the dynamics of BA cations exhibit a mix of ordered and disordered characteristics. The ordered dynamic motion disappears as the temperature continues to rise, completing the order–disorder transition. It is noted that the longest characteristic relaxation time scale for BA backbone bonds is around 20 ns. Therefore, the cooling rate used in the simulated annealing (Figure S1) should be sufficient to capture the essential dynamics and phase transition mechanism.

### Rotational Dynamics of MA Cations

In the 2D perovskite structure of (BA)_2_(MA)Pb_2_I_7_, unlike its 3D counterpart, the rotational dynamics of MA cations is influenced not just by the Pb–I cages but also by the BA layers that encapsulate the MA cations from both the top and bottom. The interaction between BA and MA cations restricts the motion of MA cations, especially in the direction perpendicular to the inorganic layers, compared to the in-plane directions. This results in oval-shaped trajectories for the MA cations, as illustrated in Figure 6g. A representative relative orientation of MA and BA cations is shown in Figure 6h. This observation aligns with the findings from Fridriksson et al.,^17^ who also noted the reduced presence of MA cations oriented perpendicular to the inorganic layers within the temperature range of 50 to 300 K.

The polarization of MA cations exhibits notable variations across different temperatures before and after the phase transition, as illustrated in Figure 6a–c. These figures present the scatter plots of nitrogen or N (in blue) and carbon or C (in red) atoms of the MA cation within the Pb–I cages at temperatures of 50, 125, and 150 K, observed over a 50 ns MD simulation. At 50 K, the average orientations of the MA dipoles on the *XY* plane predominantly point toward the corner-sharing iodides within the octahedra (additional details can be seen from Supporting Information Movie S10). This indicates that the hydrogen bonds between the  group in the MA cation and the iodides within the octahedra are asymmetric. To quantify the effects of hydrogen bonds, the average lifetimes of the hydrogen bond for both MA and BA are plotted against the temperature in Figure 6h. Here, a hydrogen bond is considered present when the distance between a hydrogen atom and an iodine atom is less than 3 Å. Notably, the lifetime of the hydrogen bond for MA at 50 K is significantly longer compared to other temperatures, indicating strong hydrogen bonding that substantially limits MA’s motion, as illustrated by the trajectories shown in Figure 6a. It is noted that the MA dipole arrangement at 50 K is similar to what was observed in 3D perovskite (MA)PbI_3_, where the MA dipoles are primarily influenced by the hydrogen bonds between the  groups of MA and the corner-sharing iodides.^18,20,21^ This suggests that the MA cations in (BA)_2_(MA)Pb_2_I_7_ are subjected to a similar hydrogen bonding environment as (MA)PbI_3_ at low temperatures.

With the temperature rising to 125 K, the lifetime of the hydrogen bond for MA significantly decreases, leading to fewer constraints on MA’s dynamics. As a result, MA exhibits an intriguing antiparallel polarization pattern, as shown in Figure 6b. This pattern is characterized by the minor offsets between the average trajectories of N and C, highlighted with yellow arrows. Interestingly, a similar antiparallel orientation is observed in the alignment of BA cations, as illustrated in Figure 6e, with green arrows indicating the head-to-tail orientation of the BA backbone. Both sets of arrows, yellow and green, are oriented toward the same direction, either +*Y* or −*Y*. This alignment suggests the role of weak vdW forces between the CH_3_ group in MA and the C backbone in BA in coordinating the orientations of BA and MA. However, at 50 K, this interaction is overshadowed by the more dominant hydrogen bonding between the  group in MA and the iodides within the cage. At 150 K, the phase transition of (BA)_2_(MA)Pb_2_I_7_ occurs, characterized by melting of the BA cations, which weakens the interaction between the BA and MA cations. Furthermore, with the increased kinetic energy of MA, their polarization patterns disappear, as shown in Figure 6c, suggesting their quasi-liquid behavior at higher temperatures, similar to the behavior observed in (MA)PbI_3_ at and above its phase transition temperature.^20^

To explore the impact of the rotational dynamics of MA cations on the phase transition, we conducted an MD simulation with the rotational freedoms of their N–C bonds deliberately constrained. It was found that under these restrictions, the phase transition shifted to a slightly higher temperature of 155 K. The process of such a phase transition was captured in Supporting Information Movie S11. This indicates that while the melting of BA cations primarily drives the phase transition, the rotational dynamics of MA cations also contribute to the transition, likely through the interactions between BA and MA cations as discussed above. It is noteworthy that a previous experiment by Dahod et al.^6^ showed that replacing MA with FA cation in 2D perovskites leads to a phase transition occurring at a temperature approximately 15 K lower. Our findings suggest that this difference in the critical phase transition temperature may be attributed to the distinct rotational dynamics of the cations, influenced by their different hydrogen bonding interactions with the iodides.

### Hydrogen Bonding

The preceding section demonstrates the role of hydrogen bonding between MA cations and the iodides within the cage on the phase transition of (BA)_2_(MA)Pb_2_I_7_, particularly through its impact on MA’s rotational dynamics. This section shows that hydrogen bonding between BA cations and iodides also significantly contributes to the phase transition process.

As shown in Figure 6h, the hydrogen bonds are formed between the  group of BA cations and the iodides at the terminal Pb–I bonds and those at the edges of the cage in proximity to the BA. These hydrogen bonds frequently break and reform due to the rotational movement of the N–H bonds in  group. Notably, the lifetime of these hydrogen bonds significantly decreases during the phase transition from 1093 ps at 50 K to just 52 ps at 150 K. To assess the influence of this hydrogen bonding on the phase transition, we performed an MD simulation where the rotational freedom of N–H bonds in the  group was deliberately restricted. This simulated a scenario of extremely stable hydrogen bonds, preventing any spin and conical rotation of the  group. With these constraints, the system exhibited no phase transition when heated from 125 to 200 K (see Supporting Information Movie S12).

Additionally, our MD simulations reveal an anisotropic hydrogen bonding network between BA cations and iodides, as illustrated in Figure 6d–f, which displays the projected trajectories (in cyan) of the N atom in the  group of BA across different temperatures. At the low-temperature phase of 50 and 125 K, denser trajectories are observed near the corners of the cage, areas highlighted by transparent rectangles with a red tint. This suggests that the  group is more likely to be found in these regions due to more persistent hydrogen bonding between the  group and the iodides close to the corner. By contrast, in the high-temperature phase at 150 K, denser N trajectories are noticeable near the edges of the cage, suggesting more persistent hydrogen bonding between the  group and the iodides close to the edge. As will be shown in a subsequent section, this shift in the hydrogen bonding pattern between BA cations and iodides within the octahedra leads to distinct octahedral distortions.

Our results highlight the significant influence of hydrogen bonding between both MA and BA cations and the iodides on the phase transition. This suggests that the phase transition process could be modulated by adjusting the strength of these hydrogen bonds through strategic design of organic cations and the inorganic layer.

### Octahedra Distortion

**Figure 7:**
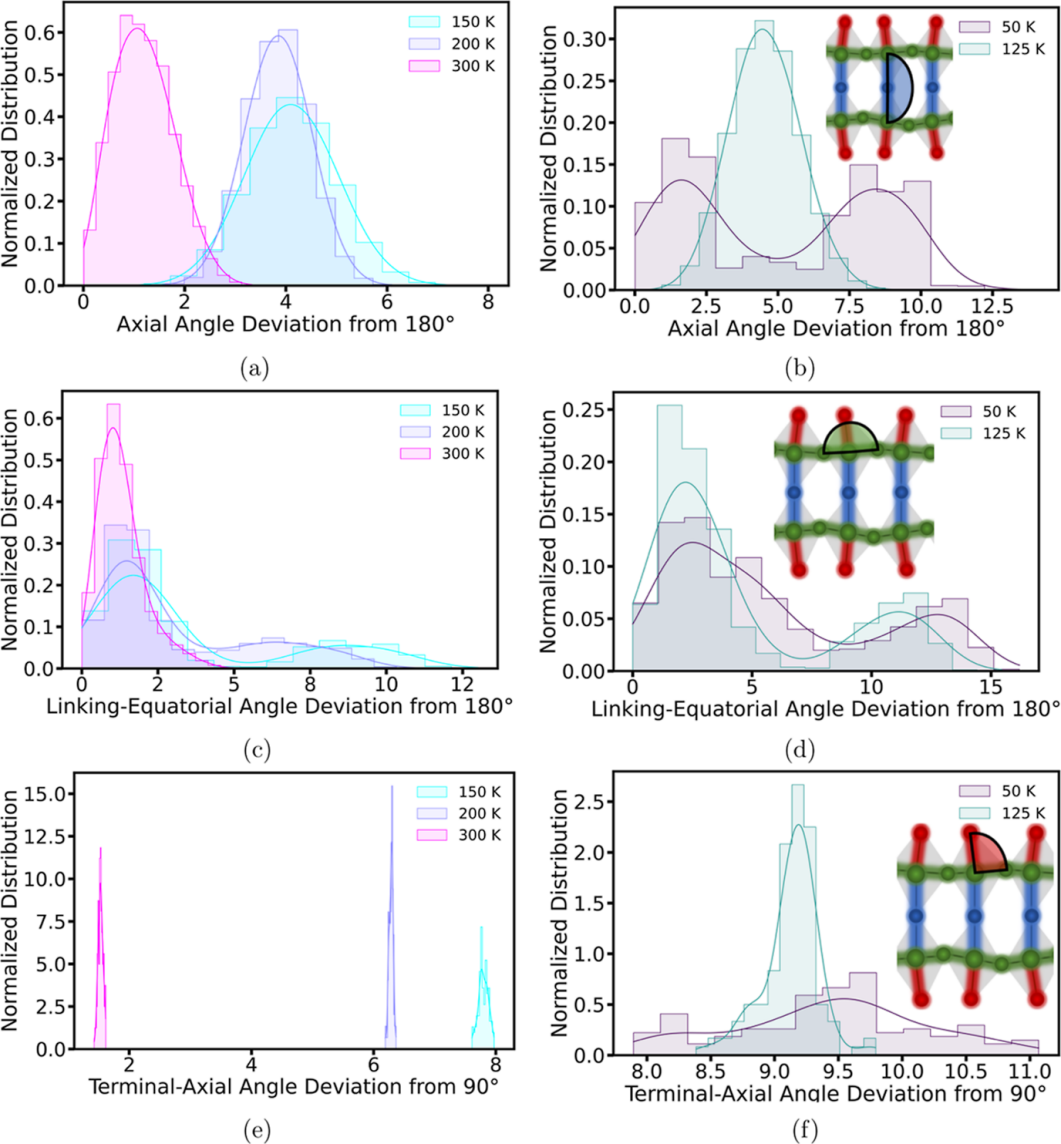
Normalized distributions of (a,b) axial Pb–I–Pb bond angle deviation from 180°, (c,d) linking-equatorial I–Pb–I bond angle deviation from 180°, and (e,f) terminal-axial Pb–I bond angle deviation from 90°. The right panels correspond to the triclinic crystal system.

Previous experiments^8,16^ have shown that the octahedra within the inorganic layer in 2D perovskites exhibit structural distortions in both low- and high-temperature phases. Such distortion was considered the displacive characteristic of the phase transition, mixed with the order–disorder transition mechanism.^16^ This section focuses on analyzing the octahedra distortions obtained from our MD simulation, aiming to uncover the mechanisms governing these distortions. We quantify the distortions using two specific tilting angles: equatorial Pb–I–Pb angle and axial Pb–I–Pb angle, as schematically shown in Figure 7b,d, respectively. In addition, the terminal Pb–I bond tilting angle, as shown in Figure 7f, quantifies the terminal bond’s tilting, attributable to the interactions between organic BA cations and the octahedra. Given the significant variation in distortion among different octahedra, we present the distribution of these tilting angles to provide a more comprehensive understanding of the structural changes.

Octahedral distortion in 2D perovskites may come from two primary interactions: the hydrogen bonding between octahedra and BA cations and, similarly, between octahedra and MA cations. By contrast, 3D perovskites, lacking an organic layer, depend solely on the interaction between MA cations and octahedra. Previous research has indicated that in 3D perovskites, octahedral tilting angles increase continuously with temperature.^21^ However, our findings show an opposite trend with a decrease in the average octahedral tilting angles, both equatorial and axial, as temperature rises, which is consistent with previous experimental observations on 2D perovskites.^16^ Such a difference suggests that the interactions between the organic layer and octahedra play a more dominant role in octahedral tilting in 2D perovskites. Evidence of this is seen in the tilting of the terminal Pb–I bonds, which increases as the temperature decreases, as shown in Figure 7e,f. The terminal bond tilting is primarily driven by the hydrogen bond interactions between terminal iodides and BA cations, which then propagate distortion through the octahedra. Across the phase transition temperature, a notable change can be observed in the histogram of the terminal Pb–I bond tilting. This is mainly due to the significant shear deformation transferred from the organic to the inorganic layer during phase transition, as shown in Figure 3.

As discussed in the previous section, the hydrogen bonding between BA cations and iodides forms an anisotropic network, illustrated in Figure 6d–f. This anisotropic arrangement of hydrogen bonds causes the edge Pb–I–Pb bonds in the octahedra to undergo differential hydrogen bond interactions, resulting in different tilting of the bond angles. Specifically, in Figure 6d–f, two edges highlighted by a red tint are subject to stronger hydrogen bond interactions compared with the other two edges. At low temperatures, the increased lifetime of these hydrogen bonds leads to more significant octahedral distortions. Moreover, this anisotropic behavior results in a bimodal distribution of octahedral tilting angles at lower temperatures, with angles separating into two groups, as demonstrated in Figure 7b,d, where the edges under stronger hydrogen bonding show greater tilting angles. When temperature increases, the reduction in hydrogen bonding strength diminishes or eliminates this bifurcation. In fact, Menahem et al.^16^ have previously postulated an anisotropic hydrogen bonding network to explain the equatorial octahedral tilting observed in their experimental studies on the (BA)_2_PbI_4_ across both low- and high-temperature phases. In addition, the phenomenon of equatorial tilting angle bifurcation was similarly noted in the experiment by Paritmongkol et al.^8^ Our results not only corroborate earlier observations and hypotheses but also clarify the driving mechanisms.

## Conclusions

In this study, all-atom MD simulations were carried out to investigate the temperature-induced phase transition in (BA)_2_(MA)Pb_2_I_7_. Our simulations predicted the transition from a triclinic phase at lower temperatures to a tetragonal phase at higher temperatures, with the predicted structures closely aligning with those measured in experiments. The critical phase transition temperature predicted by our simulations is lower than that observed experimentally. Further work is certainly required to enhance the accuracy of the empirical force field, possibly through reparametrization of the force field parameters or the use of machine learning potentials. Nevertheless, many predictions from our simulations are supported by the existing experimental data. Some of these predictions are qualitative, while others also demonstrate quantitative agreement. Beyond the order–disorder phase transition mechanism, our study uncovers the phenomena and atomic-level mechanisms contributing to the phase transition, summarized as follows.

First, our simulations enhance the understanding of the order–disorder transition mechanism, marked by the evolving rotation dynamics of BA cations during phase transition. In the low-temperature phase, BA cations are orderly arranged, featuring tilted and intercalated alkylammonium chains. With rising temperatures, the N–H bonds in BA initially melt, followed by the melting of the tail bonds in the BA backbone, initiating the phase transition. Further heating amplifies the dynamics of BA cations, leading to a patterned, synchronized conical pendulum-like swinging motion displaying a mix of ordered and disordered behaviors. The ordered dynamics cease when BA cations are fully melted, marking a complete transition from the ordered to a disordered state.

Second, our simulations reveal that MA cations exhibit distinct polarization patterns across the phase transition. At low temperatures, MA dipoles primarily orient toward the corner-sharing iodides due to asymmetric hydrogen bonding between MA and the iodides. Close to phase transition, MA cations display an antiparallel polarization pattern due to BA–MA interactions. Beyond the phase transition temperature, the polarization pattern of MA vanishes due to melting of MA and BA cations. Additionally, the simulations show that constraining the rotation of MA cations increases the phase transition temperature, indicating the role of MA in the phase transition through interactions with BA cations.

Third, our simulation uncovers an anisotropic hydrogen bonding network between BA cations and iodides within octahedra, which is pivotal for both the melting of BA cations and the distortion of the octahedra. At lower temperatures, distortions are more pronounced due to the extended lifetime of hydrogen bonds, and a significant variance in distortions among octahedra is observed due to the anisotropic nature of the hydrogen bonding network. These findings offer a mechanistic understanding of the displacive characteristics of the phase transition reported in earlier experiments. Finally, due to the model size used in the current work, the phase nucleation and growth mechanisms, including the effects of defects, are not explored, which could be an interesting topic for future research.

## Methods

Molecular dynamics simulations were conducted using the Large-scale Atomic/Molecular Massively Parallel Simulator (LAMMPS),^22^ with system construction facilitated by Moltemplate.^23^ Post-simulation analyses and visualization were performed using OVITO Pro.^24^

We utilized Fridriksson et al.’s force field^17^ to capture the complex atom interactions within the (BA)_2_(MA)Pb_2_I_7_ system. The model used by Fridriksson is derived from the MYP potential,^20,25^ which has been adopted to study 2D perovskites.^26,27^ Simulations incorporated the particle–particle particle–mesh (PPPM) methodology for long-range Coulombic interactions, ensuring a force accuracy of 10^–7^.^28^ Notably, we recalculated the PPPM grid size every 50 ps to address the triclinic cell’s varying tilt, thus preserving simulation precision.

A full periodic supercell was constructed by replicating the experimentally obtained CIF file^29^ four times along both the *X* and *Y* axes, as shown in Figure 1. This ensured that the supercell dimensions of 3.6 × 3.5 × 3.9 nm were slightly larger than twice the cutoff distance of 17 Å for both Lennard-Jones and Coulombic interactions. The supercell underwent energy and force minimizations, including volumetric relaxation.

To achieve relaxed structures at various temperatures, we employed an annealing process. This process began by generating a velocity ensemble mimicking thermal atomic vibrations at 400 K using a Gaussian probability distribution. The velocity-Verlet integration algorithm, with a 1 fs time step, was utilized for the successive numerical integration of Newton’s equations of motion. We attained equilibrium at 400 K over 30 ns using the isothermal–isobaric (*NPT*) ensemble method, incorporating a Nose–Hoover thermostat and barostat algorithms. Notably, the barostat was applied to all stress components, maintaining their values at zero throughout the simulation. For each time step, we adjusted temperature and pressure, setting their relaxation times to 100 and 1000 times the time step, respectively. To stabilize initial fluctuations in the temperature and pressure, a series of five linked thermostats and barostats were used. For enhanced accuracy in *NPT* integration, both thermostat and barostat updates were subdivided into 100 discrete steps.^30^

To constrain the rotational degrees of freedom of a specific bond in the organic cations, the two atoms forming the bond are treated as a single rigid body using the “fix rigid” command in LAMMPS. At each time step, the total force and torque on each rigid body are calculated by summing the forces and torques on its constituent particles. The coordinates, velocities, and orientations of the atoms in each rigid body are then updated accordingly, ensuring that the entire body moves and rotates as a single entity. All three torque components and all three rotational velocities are then set to zero, fully constraining the bond or rigid body’s rotation. It should be noted that it is essential to adjust the total number of degrees of freedom after creating the rigid bodies to maintain the system at the target temperature, similar to a simulation without constraints or rigid bodies.

## Supporting Information Available

MD simulation annealing procedure; time-averaged atomic configurations of (BA)_2_(MA)Pb_2_I_7_ at 1, 50, 100, 150, 200, 250, and 300 K; captions for movies 1–12; and access link for LAMMPS simulation files and Supplementary Movies shared through figshare: https://doi.org/10.6084/m9.figshare.25457815 (PDF)
