# Supplementary material for: Temperature-Induced Phase Transition in 2D Alkylammonium Lead Halide Perovskites: A Molecular Dynamics Study
Source: ACS Nano. 2024 Aug 13;18(34):22926–37. doi: 10.1021/acsnano.4c03903 (PMC11363124; doi:10.1021/acsnano.4c03903)
Supplement: Supplementary file 1 — nn4c03903_si_001.pdf [file nn4c03903_si_001.pdf]

Supplementary Information

Temperature-Induced Phase Transition in 2D Alkylammonium Lead Halide Perovskites: A Molecular Dynamics Study

Reza Namakian,[†] Maria Alejandra Garzon,[†] Qing Tu,[‡] Ali Erdemir,[†,‡] and Wei Gao*,[†,‡]

†*J. Mike Walker'66 Department of Mechanical Engineering, Texas A&M University, College Station, TX 77843, United States*

‡*Department of Materials Science & Engineering, Texas A&M University, College Station, TX 77840, United States*

E-mail: wei.gao@tamu.edu

Phone: +1 (979) 458-0250

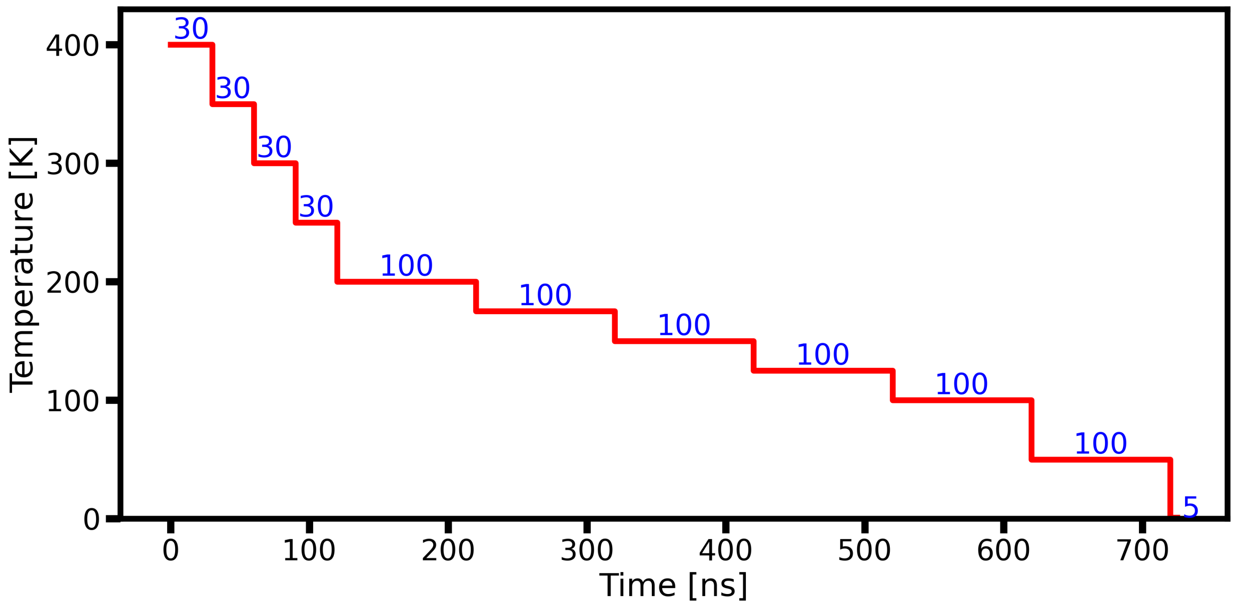

Figure S1: Molecular dynamics (MD) simulation annealing procedure illustrating the temperature reduction from 400 K to 1 K. Temperature increments are as follows: 400 → 350 → 300 → 250 → 200 → 175 → 150 → 125 → 100 → 50 → 1. The indicated blue values atop each segment represent the corresponding relaxation time applied at the specific temperature point.

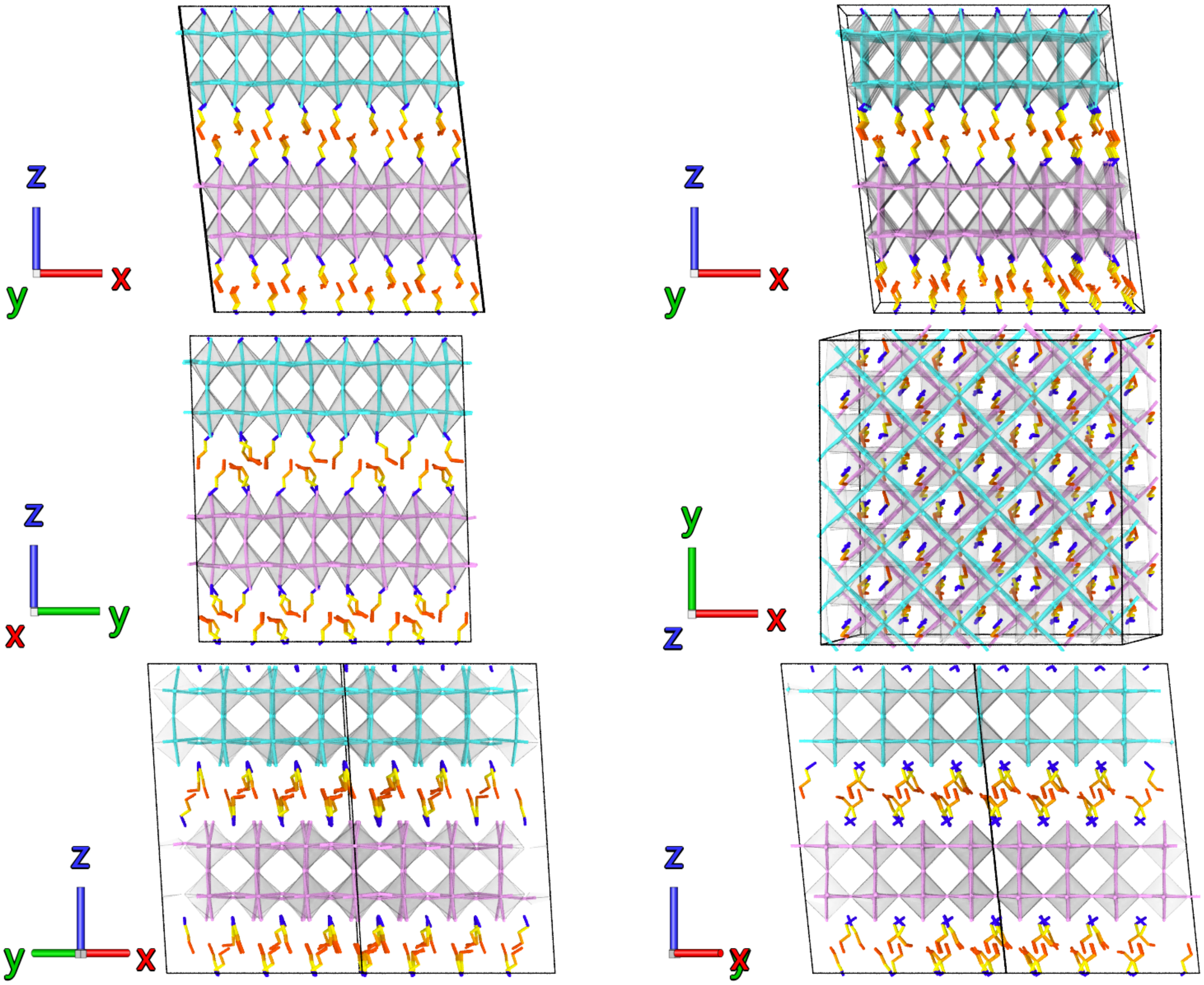

Figure S2: Time-averaged atomic configurations of $(BA)_2(MA)Pb_2I_7$ at 1 K, obtained from a centered 200 ps interval around the respective snapshot. Distinct color schemes used for BA backbone bonds and Pb-I bonds in upper and lower inorganic layers to enhance visualization. The octahedral geometry is depicted in translucent white, and MA cations are omitted.

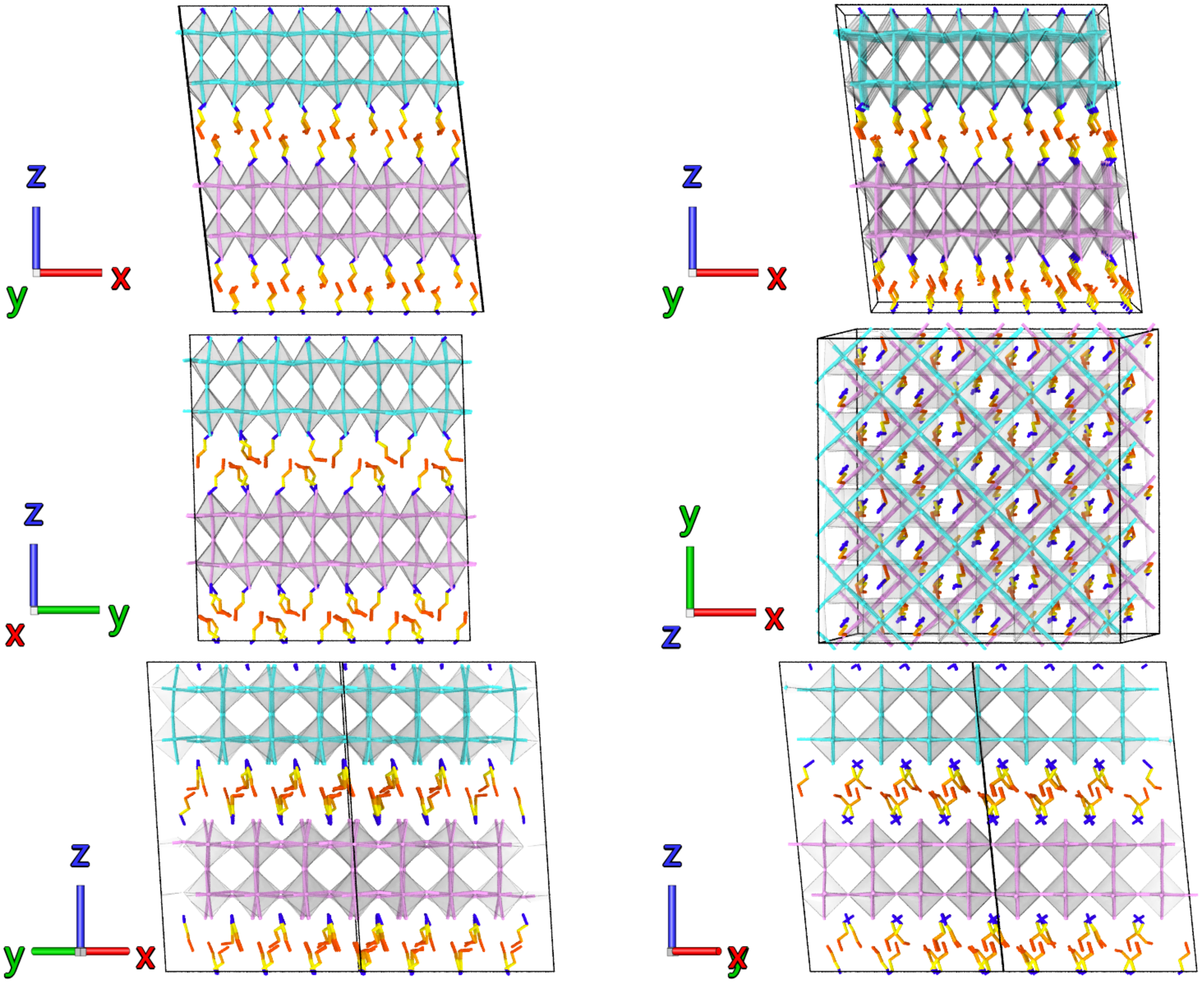

Figure S3: Time-averaged atomic configurations of $(BA)_2(MA)Pb_2I_7$ at 50 K, obtained from a centered 200 ps interval around the respective snapshot. Distinct color schemes used for BA backbone bonds and Pb-I bonds in upper and lower inorganic layers to enhance visualization. The octahedral geometry is depicted in translucent white, and MA cations are omitted.

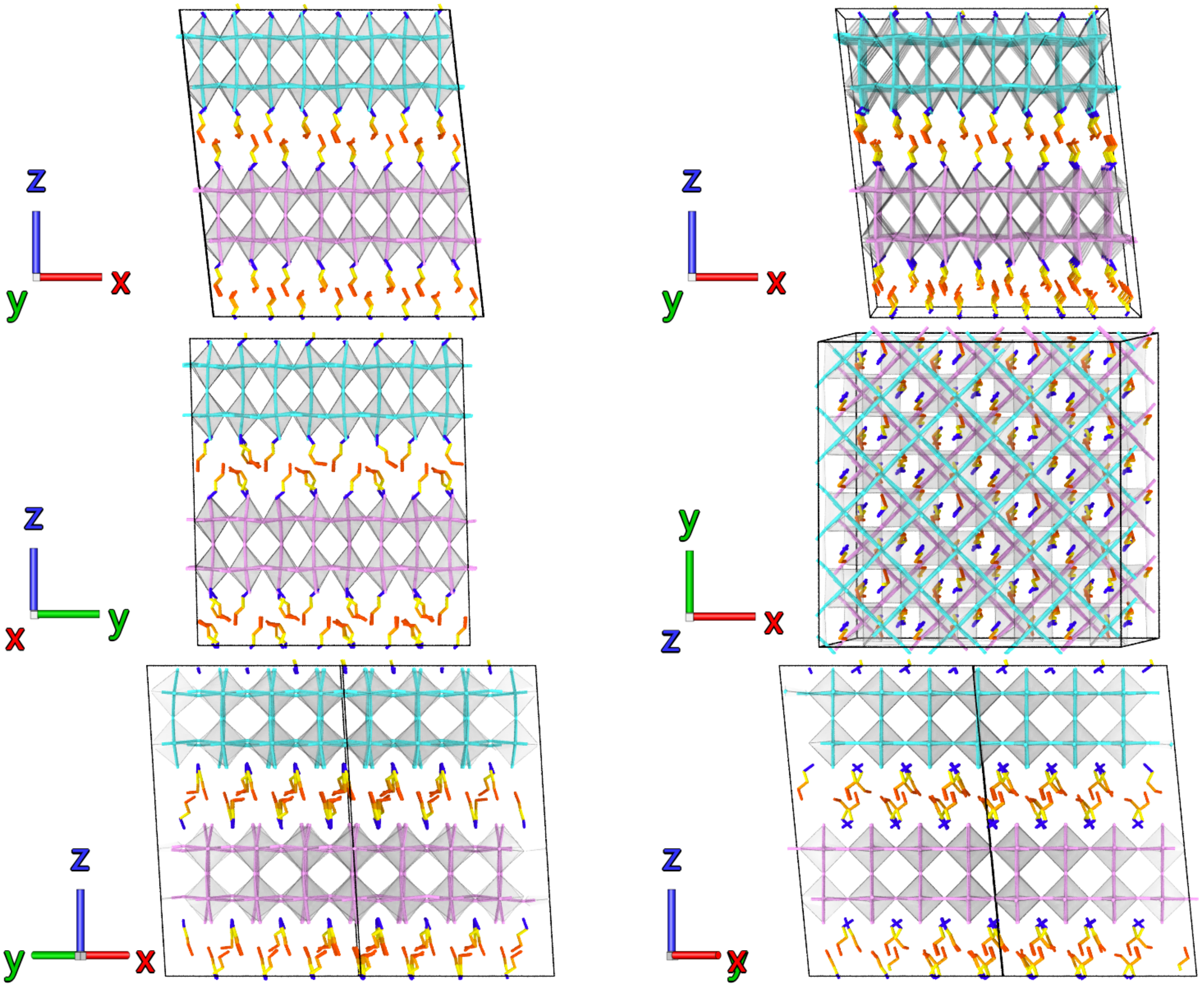

Figure S4: Time-averaged atomic configurations of $(BA)_2(MA)Pb_2I_7$ at 100 K, obtained from a centered 200 ps interval around the respective snapshot. Distinct color schemes used for BA backbone bonds and Pb-I bonds in upper and lower inorganic layers to enhance visualization. The octahedral geometry is depicted in translucent white, and MA cations are omitted.

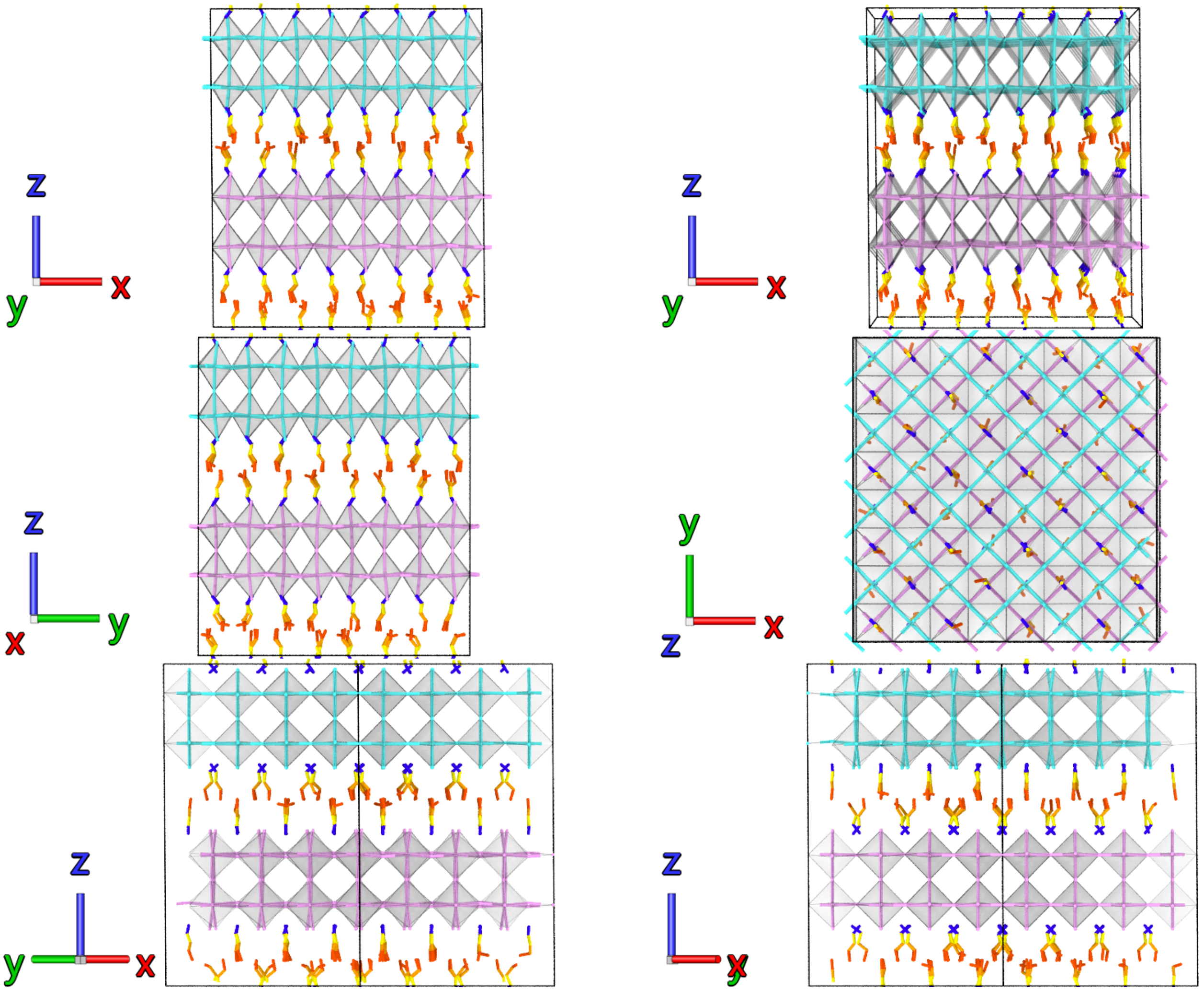

Figure S5: Time-averaged atomic configurations of $(BA)_2(MA)Pb_2I_7$ at 150 K, obtained from a centered 200 ps interval around the respective snapshot. Distinct color schemes used for BA backbone bonds and Pb-I bonds in upper and lower inorganic layers to enhance visualization. The octahedral geometry is depicted in translucent white, and MA cations are omitted.

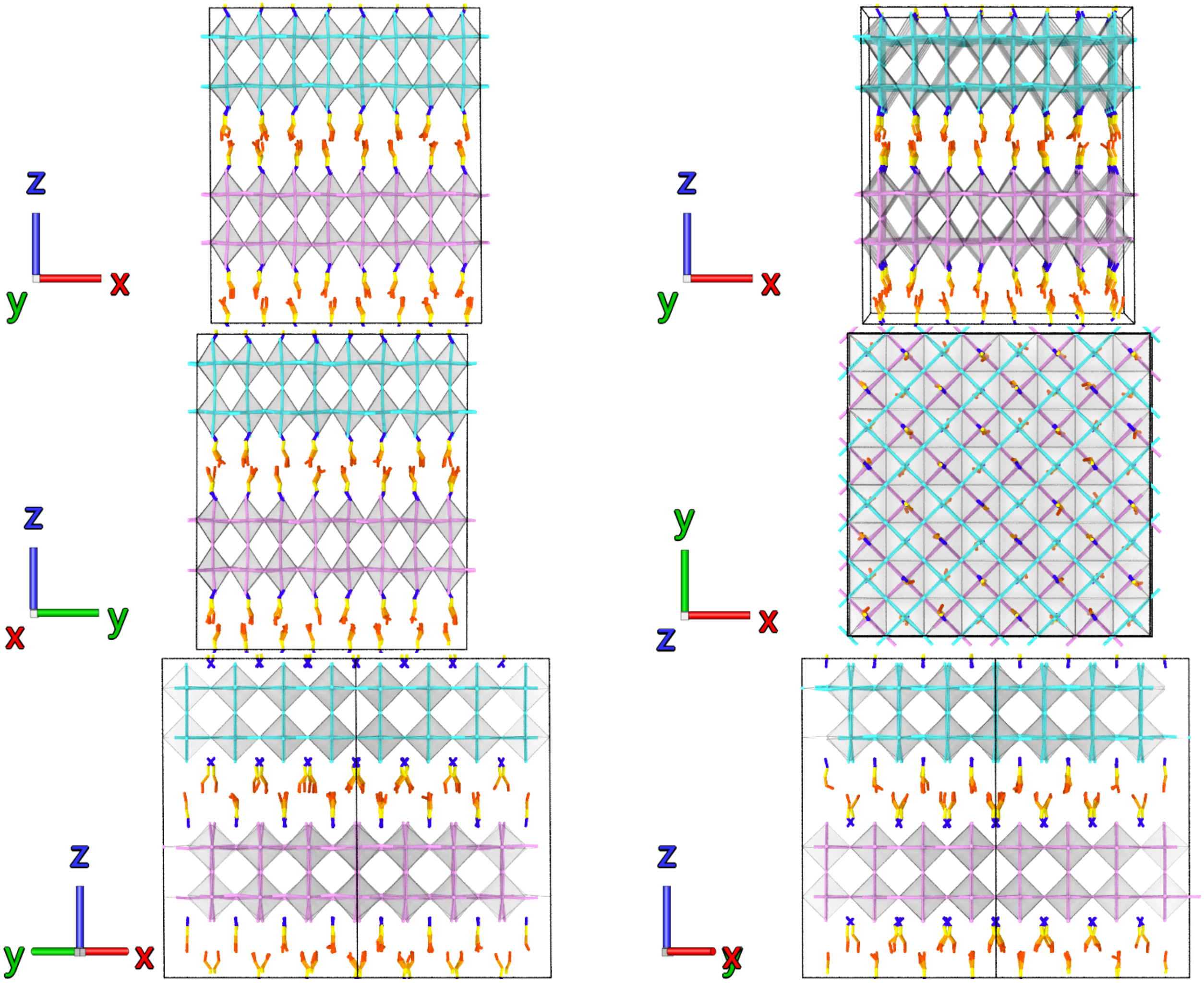

Figure S6: Time-averaged atomic configurations of $(BA)_2(MA)Pb_2I_7$ at 200 K, obtained from a centered 200 ps interval around the respective snapshot. Distinct color schemes used for BA backbone bonds and Pb-I bonds in upper and lower inorganic layers to enhance visualization. The octahedral geometry is depicted in translucent white, and MA cations are omitted.

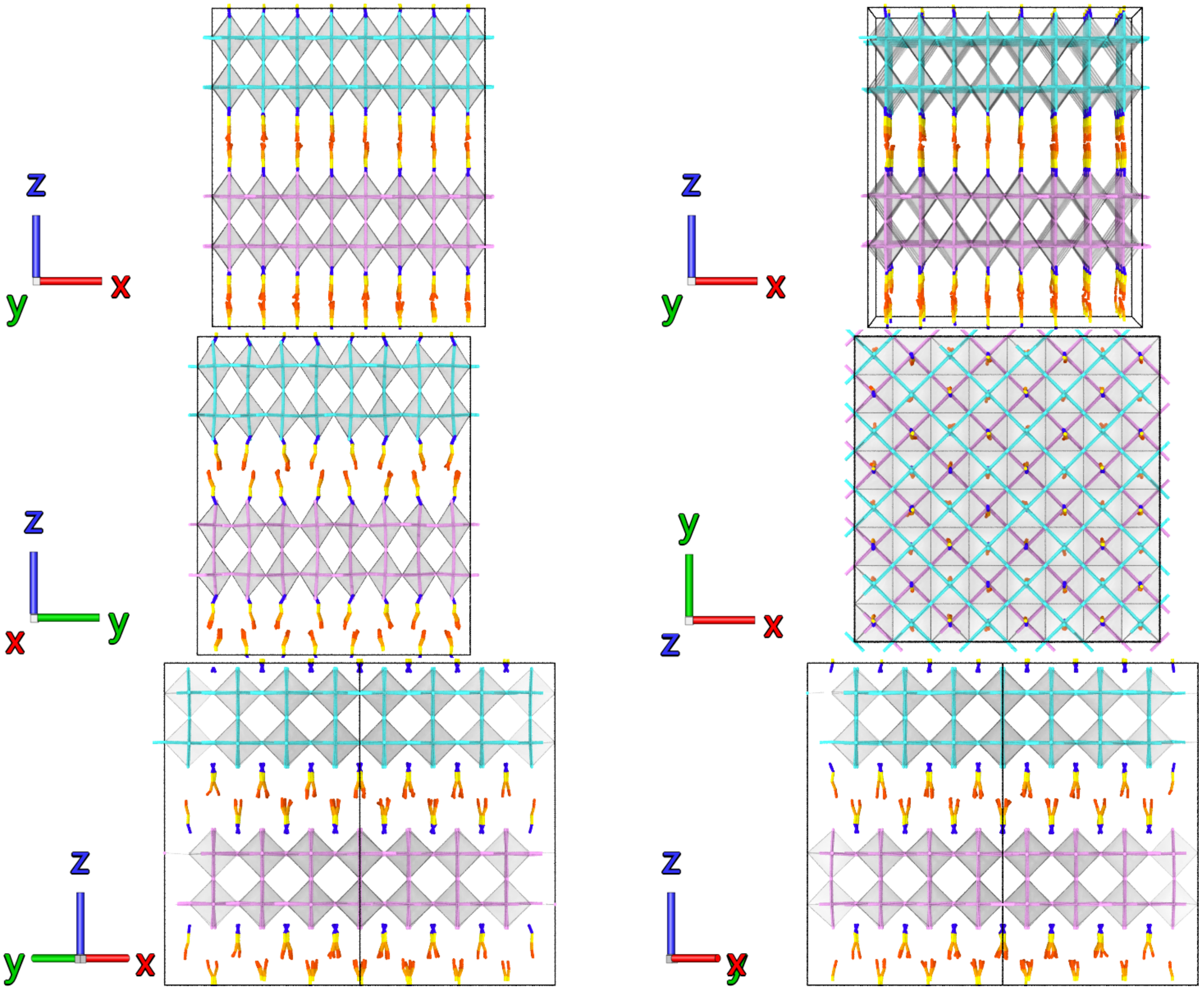

Figure S7: Time-averaged atomic configurations of $(BA)_2(MA)Pb_2I_7$ at 250 K, obtained from a centered 200 ps interval around the respective snapshot. Distinct color schemes used for BA backbone bonds and Pb-I bonds in upper and lower inorganic layers to enhance visualization. The octahedral geometry is depicted in translucent white, and MA cations are omitted.

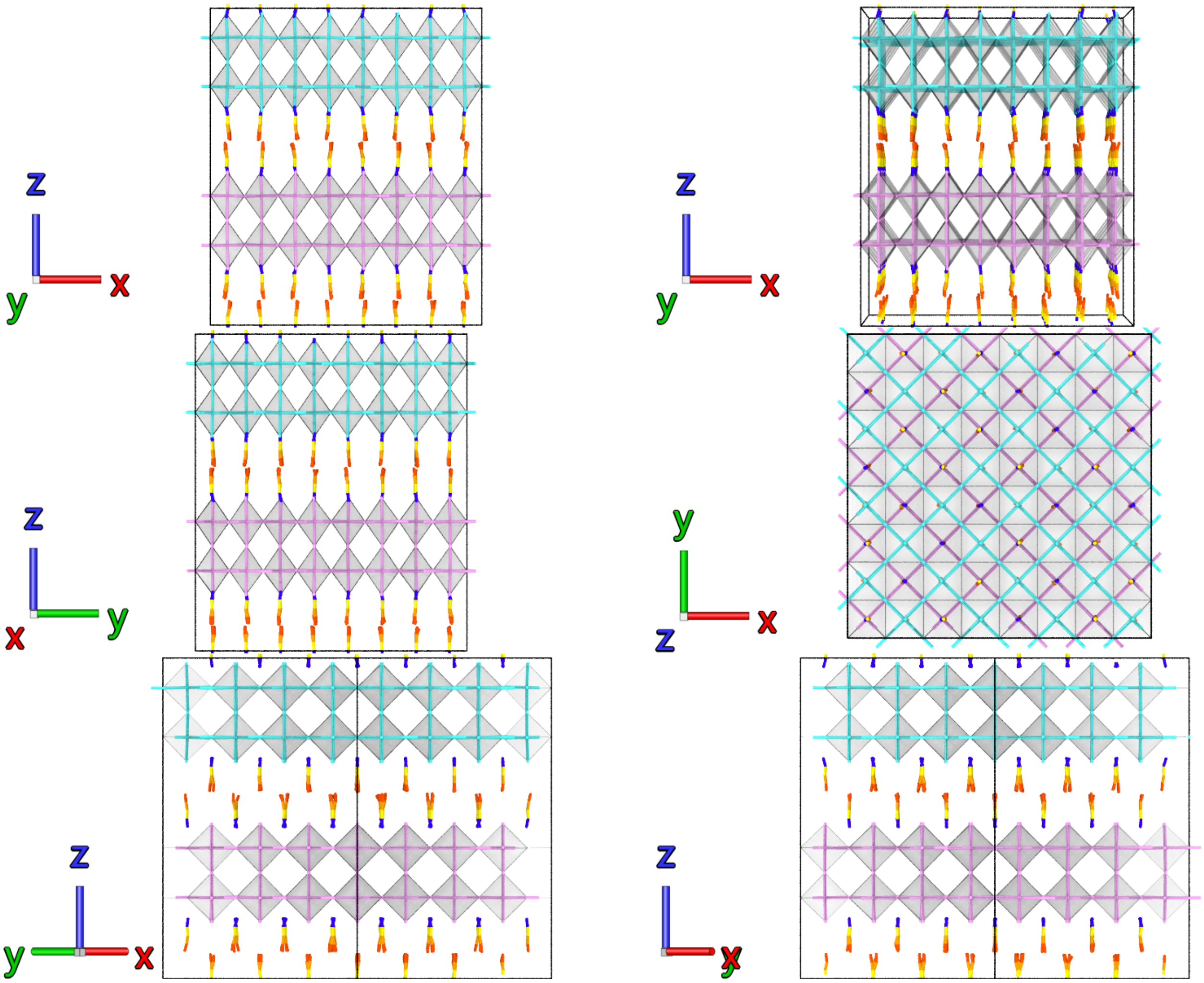

Figure S8: Time-averaged atomic configurations of $(BA)_2(MA)Pb_2I_7$ at 300 K, obtained from a centered 200 ps interval around the respective snapshot. Distinct color schemes used for BA backbone bonds and Pb-I bonds in upper and lower inorganic layers to enhance visualization. The octahedral geometry is depicted in translucent white, and MA cations are omitted.

Supplementary Movies

Moives are shared through figshare.com and can be accessed using the following link:

https://doi.org/10.6084/m9.figshare.25457815

The captions of the movies are provided as follow:

Supplementary Movie 1.

Time-averaged atomic positions during 20 ns simulated phase transition of $(BA)_2(MA)Pb_2I_7$, wherein the temperature is increased from 125 K to 150 K. To enhance clarity, atomic oscillations were minimized by averaging 200 consecutive simulation trajectories, yielding a representation based on a 200 ps interval per snapshot. Pb-I bonds in the inorganic layers are distinguished by color: cyan for the upper layer and pink for the lower. The octahedral geometry, with centrally located Pb, is displayed in translucent white. Due to the accelerated rotation of MA cations at this temperature, their time-averaged conformations are indiscernible and thus omitted from the visualization. The hydrogen bonds in organic cations are not displayed in these snapshots.

Supplementary Movie 2.

Time-averaged atomic positions over a 20 ns simulation of $(BA)_2(MA)Pb_2I_7$, wherein the temperature is increased from 125 K to 200 K, and the rotational degrees of freedom of C_3-C_4 bond in BA cations remain constrained. To enhance clarity, atomic oscillations were minimized by averaging 200 consecutive simulation trajectories, yielding a representation based on a 200 ps interval per snapshot. Pb-I bonds in the inorganic layers are distinguished by color: cyan for the upper layer and pink for the lower. The octahedral geometry, with centrally located Pb, is displayed in translucent white. Due to the accelerated rotation of MA cations at this temperature, their time-averaged conformations are indiscernible and thus omitted from the visualization. The hydrogen bonds in organic cations are not displayed in these snapshots.

Supplementary Movie 3.

Time-averaged atomic positions of $(BA)_2(MA)Pb_2I_7$ during simulated annealing. This visualization reveals the dynamics of $(BA)_2(MA)Pb_2I_7$ under simulated annealing from 10-30 ns at 300 K. To enhance clarity, atomic oscillations were minimized by averaging 200 consecutive simulation trajectories, yielding a representation based on a 200 ps interval per snapshot. Pb-I bonds in the inorganic layers are distinguished by color: cyan for the upper layer and pink for the lower. The octahedral geometry, with centrally located Pb, is displayed in translucent white. Due to the accelerated rotations of MA cations at this temperature, their time-averaged conformations are indiscernible and thus omitted from the visualization. The hydrogen bonds in organic cations are not displayed in these snapshots.

Supplementary Movie 4.

Time-averaged atomic positions of $(BA)_2(MA)Pb_2I_7$ during simulated annealing. This visualization reveals the dynamics of $(BA)_2(MA)Pb_2I_7$ under simulated annealing from 10-30 ns at 250 K. To enhance clarity, atomic oscillations were minimized by averaging 200 consecutive simulation trajectories, yielding a representation based on a 200 ps interval per snapshot. Pb-I bonds in the inorganic layers are distinguished by color: cyan for the upper layer and pink for the lower. The octahedral geometry, with centrally located Pb, is displayed in translucent white. Due to the accelerated rotation of MA cations at this temperature, their time-averaged conformations are indiscernible and thus omitted from the visualization. The hydrogen bonds in organic cations are not displayed in these snapshots.

Supplementary Movie 5.

Time-averaged atomic positions of $(BA)_2(MA)Pb_2I_7$ during simulated annealing. This visualization reveals the dynamics of $(BA)_2(MA)Pb_2I_7$ under simulated annealing from 80-100 ns at 200 K. To enhance clarity, atomic oscillations were minimized by averaging 200 consecutive simulation trajectories, yielding a representation based on a 200 ps interval per

snapshot. Pb-I bonds in the inorganic layers are distinguished by color: cyan for the upper layer and pink for the lower. The octahedral geometry, with centrally located Pb, is displayed in translucent white. Due to the accelerated rotation of MA cations at this temperature, their time-averaged conformations are indiscernible and thus omitted from the visualization. The hydrogen bonds in organic cations are not displayed in these snapshots.

Supplementary Movie 6.

Time-averaged atomic positions of $(BA)_2(MA)Pb_2I_7$ during simulated annealing. This visualization reveals the dynamics of $(BA)_2(MA)Pb_2I_7$ under simulated annealing from 80-100 ns at 175 K. To enhance clarity, atomic oscillations were minimized by averaging 200 consecutive simulation trajectories, yielding a representation based on a 200 ps interval per snapshot. Pb-I bonds in the inorganic layers are distinguished by color: cyan for the upper layer and pink for the lower. The octahedral geometry, with centrally located Pb, is displayed in translucent white. Due to the accelerated rotation of MA cations at this temperature, their time-averaged conformations are indiscernible and thus omitted from the visualization. The hydrogen bonds in organic cations are not displayed in these snapshots.

Supplementary Movie 7.

Time-averaged atomic positions of $(BA)_2(MA)Pb_2I_7$ during simulated annealing. This visualization reveals the dynamics of $(BA)_2(MA)Pb_2I_7$ under simulated annealing from 80-100 ns at 150 K. To enhance clarity, atomic oscillations were minimized by averaging 200 consecutive simulation trajectories, yielding a representation based on a 200 ps interval per snapshot. Pb-I bonds in the inorganic layers are distinguished by color: cyan for the upper layer and pink for the lower. The octahedral geometry, with centrally located Pb, is displayed in translucent white. Due to the accelerated rotation of MA cations at this temperature, their time-averaged conformations are indiscernible and thus omitted from the visualization. The hydrogen bonds in organic cations are not displayed in these snapshots.

Supplementary Movie 8.

Time-averaged atomic positions of $(BA)_2(MA)Pb_2I_7$ during simulated annealing. This visualization reveals the dynamics of $(BA)_2(MA)Pb_2I_7$ under simulated annealing from 80-100 ns at 125 K. To enhance clarity, atomic oscillations were minimized by averaging 200 consecutive simulation trajectories, yielding a representation based on a 200 ps interval per snapshot. Pb-I bonds in the inorganic layers are distinguished by color: cyan for the upper layer and pink for the lower. The octahedral geometry, with centrally located Pb, is displayed in translucent white. Due to the accelerated rotation of MA cations at this temperature, their time-averaged conformations are indiscernible and thus omitted from the visualization. The hydrogen bonds in organic cations are not displayed in these snapshots.

Supplementary Movie 9.

Time-averaged atomic positions of $(BA)_2(MA)Pb_2I_7$ during simulated annealing. This visualization reveals the dynamics of $(BA)_2(MA)Pb_2I_7$ under simulated annealing from 80-100 ns at 100 K. To enhance clarity, atomic oscillations were minimized by averaging 200 consecutive simulation trajectories, yielding a representation based on a 200 ps interval per snapshot. Pb-I bonds in the inorganic layers are distinguished by color: cyan for the upper layer and pink for the lower. The octahedral geometry, with centrally located Pb, is displayed in translucent white. Due to the accelerated rotation of MA cations at this temperature, their time-averaged conformations are indiscernible and thus omitted from the visualization. The hydrogen bonds in organic cations are not displayed in these snapshots.

Supplementary Movie 10.

Time-averaged atomic positions over a 20 ns simulation of $BA_2MAPb_2I_7$, wherein the temperature is kept at 50 K. To enhance clarity, atomic oscillations were minimized by averaging 10 consecutive simulation trajectories, yielding a representation based on a 10 ps interval per snapshot. Pb-I bonds in the inorganic layers are distinguished by color: cyan for the

upper layer and pink for the lower. The octahedral geometry, with centrally located Pb, is displayed in translucent white. BA cations are omitted from the visualization. The MA N-C bonds are depicted in blue at higher Z-positions, while the red-colored N-C bonds occupy lower Z-positions. The hydrogen bonds in organic cations are not displayed in these snapshots.

Supplementary Movie 11.

Time-averaged atomic positions over a 20 ns simulation of $(BA)_2(MA)Pb_2I_7$, wherein the temperature is increased from 125 K to 155 K, and the rotational degrees of freedom of N-C bonds in MA cations remain constrained. To enhance clarity, atomic oscillations were minimized by averaging 200 consecutive simulation trajectories, yielding a representation based on a 200 ps interval per snapshot. Pb-I bonds in the inorganic layers are distinguished by color: cyan for the upper layer and pink for the lower. The octahedral geometry, with centrally located Pb, is displayed in translucent white. MA cations are omitted from the visualization. The hydrogen bonds in organic cations are not displayed in these snapshots.

Supplementary Movie 12.

Time-averaged atomic positions over a 20 ns simulation of $(BA)_2(MA)Pb_2I_7$, wherein the temperature is increased from 125 K to 200 K, and the rotational degrees of freedom of N-H bonds within the ammonium head groups of BA cations remain constrained. To enhance clarity, atomic oscillations were minimized by averaging 200 consecutive simulation trajectories, yielding a representation based on a 200 ps interval per snapshot. Pb-I bonds in the inorganic layers are distinguished by color: cyan for the upper layer and pink for the lower. The octahedral geometry, with centrally located Pb, is displayed in translucent white. Due to the accelerated rotation of MA cations at this temperature, their time-averaged conformations are indiscernible and thus omitted from the visualization. The hydrogen bonds in

organic cations are not displayed in these snapshots.

LAMMPS files

The LAMMPS simulation files can be accessed using the following link:

https://doi.org/10.6084/m9.figshare.25457815
